# The value of total caudate lobe resection for hilar cholangiocarcinoma: a systematic review

**DOI:** 10.1097/JS9.0000000000000795

**Published:** 2023-09-21

**Authors:** Da Wang, Fei Xiong, Guanhua Wu, Qi Wang, Junsheng Chen, Wenzheng Liu, Bing Wang, Yongjun Chen

**Affiliations:** Department of Biliary-Pancreatic Surgery, Tongji Hospital, Tongji Medical College, Huazhong University of Science and Technology, Wuhan, Hubei Province, People’s Republic of China

**Keywords:** caudate lobe resection, Hilar cholangiocarcinoma, R0 resection

## Abstract

Hilar cholangiocarcinoma (HCCA) is widely considered to have a poor prognosis. In particular, combined caudate lobe resection (CLR) as a strategy for radical resection in HCCA is important for improving the R0 resection rate. However, the criteria for R0 resection, necessity of CLR, optimal extent of hepatic resection, and surgical approach are still controversial. This review aimed to summarize the findings and discuss the controversies surrounding CLR. Numerous clinical studies have shown that combined CLR treatment for HCCA improves the R0 resection rate and postoperative survival time. Whether surgery for Bismuth type I or II is combined with CLR depends on the pathological type. Considering the anatomical factors, total rather than partial CLR is recommended to achieve a higher R0 resection rate. In the resection of HCCA, a proximal ductal margin greater than or equal to 10 mm should be achieved to obtain a survival benefit. Although there is no obvious boundary between the right side (especially the paracaval portion) and the right posterior lobe of the liver, Peng’s resection line can serve as a reference marker for right-sided resection. Laparoscopic resection of the caudate lobe may be safer, more convenient, accurate, and minimally invasive than open surgery, but it needs to be completed by experienced laparoscopic doctors.

## Introduction

Highlights

- Combined with caudate lobe resection (CLR) in the treatment of hilar cholangiocarcinoma improved the R0 resection rate and the postoperative survival time.
- Considering the anatomical factors, in order to achieve a higher R0 resection rate, total CLR is recommended rather than partial CLR.
- In patients with nodular or invasive tumor, right/left hepatic lobectomy combined with CLR are recommended. For papillary type, bile duct resection combined with partial liver resection (based on the resection margin) is enough.
- LRRHCCA combined with total CLR is more convenient, with clearer vision, a better angle, and relatively small trauma than open surgery, but the operation time is relatively long and has high technical requirements.

Hilar cholangiocarcinoma (HCCA), or Klatskin tumor, is the most common malignant tumor of the biliary tract and originates in the proximal ducts between the level of the cystic duct and secondary branches of the left and right hepatic ducts^1,2^. HCCA has a poor prognosis, with a median survival time of 29.9 months and a 10-year OS of 12.8%^3^, even though it is slow-growing and late metastasizing in nature. Because of its special and important anatomical position, patients often present with obstructive jaundice; however, abdominal pain is unremarkable^4^. In the past few years, the combination of gemcitabine and cisplatin has been the frontline standard for the treatment of HCCA, offering a median survival of ~1 year only^5,6^. Clinical studies of immune checkpoint inhibition as monotherapy have shown overall low rates of objective response (5.8%) in patients with HCCA^7^.

It is generally accepted that surgical resection is the only potentially curative therapy^8^, including bile duct resection combined with major hepatectomy, hilar lymph node dissection, and hepatic artery and portal vein resection and reconstruction if necessary^9–12^. Although radical resection combined with hepatectomy is widely used in the treatment of HCCA, the choice of surgical strategy remains controversial, especially for resection of the caudate lobe^13^. According to the position of the caudate lobe, it is closely connected with the hilar bile duct, and tumors can easily invade the caudate lobe^14^. Therefore, combined caudate lobe resection (CLR) is more conducive to achieving R0 resection^15–17^. However, owing to its proximity to the portal vein, inferior vena cava, and hepatic vein, CLR is full of risks and challenges, especially in patients with cirrhosis^18,19^. In addition, the caudate lobe is connected to the right posterior lobe of the liver without an obvious boundary^20,21^, complete removal of the caudate lobe is particularly difficult. This system review focuses on the controversies regarding CLR in HCCA.

## Methods

This study was conducted according to the Preferred Reporting Items for Systematic Reviews and Meta-analyses (PRISMA) statement^22^ (https://www.sciencedirect.com/science/article/pii/S1743919121000406?via%3Dihub/) (Supplemental Digital Content 1, http://links.lww.com/JS9/B46) and AMSTAR-2 guidelines^23^ (Supplemental Digital Content 2, http://links.lww.com/JS9/B47), and the overall AMSTAR 2 quality of this systematic review is moderate. The study was registered with the Research Registry.

To clarify the value of CLR in the treatment of HCCA, we searched PubMed to select reports published before 10 January 2023, using the term ʻcaudate lobe resectionʼ. The search strategy was developed by two reviewers and peer-reviewed by a third reviewer. The search was performed by two reviewers to avoid mistakes. The search was not restricted to language, date, or publication status. Studies that compared the effects or outcomes of CLR in HCCA surgery were included. Randomized controlled trials, nonrandomized controlled trials, and retrospective clinical or cohort studies were included in our research. Research on CLR, but not HCCA, and research on HCCA that did not target CLR were excluded. Case reports, review articles, and surveys were also excluded.

The methodological quality of the studies was assessed by two authors (D. W. and G. W.). Because of lacking of randomized controlled studies, we use the Newcastle–Ottawa Scale (NOS) to assess the quality of nonrandomized studies. The maximal score of NOS is nine scores, including the ʻSelectionʼ (four scores), ʻComparabilityʼ (two scores), and ʻOutcomeʼ (three scores). Cohort studies with a NOS score less than 6 were considered of low quality which would be excluded.

We used Review Manager 5.3 software (Cochrane Collaboration, Oxford) for the first step of statistical analyses. Risk ratios (RRs) were used to estimate the dichotomous variables. Values of *P* <0.05 supported the statistical significance of RRs. All data will be first detected heterogeneity using *χ*² test. *I*² was used to quantify heterogeneity between studies. An *I*²<25% was considered low heterogeneity, 25–50% moderate heterogeneity, and *I*²>50% considerable heterogeneity. We choose the random effects model to assess the data. We use forest plots to present the combined results.

## Results

### Data extraction

**Figure 1:**
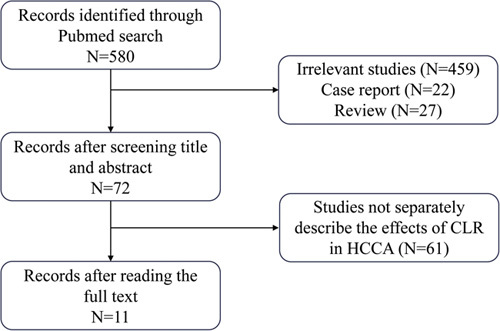
The flowchart of study selection.

A total of 580 documents were retrieved. After a screening of titles and abstracts, 459 articles were excluded for irrelevancy. In the remaining 121 studies, there are 22 articles for case reports, and 27 articles for reviews. When excluding these studies, there were 72 studies for CLR in HCCA. Then we read the full text, 61 studies were excluded as they did not separately describe the effects of CLR in HCCA. Finally, 11 studies were screened to describe in detail the treatment of HCCA combined with CLR (Fig. 1). Then we use the NOS to access the quality of these studies (Table S1, Supplemental Digital Content 3, http://links.lww.com/JS9/B48), and three studies were used for meta-analysis.

The following data were extracted: name of the first author; year of publication; period of the patient who underwent surgery; number of patients who underwent surgery; R0 resection rate; 90-day postoperative mortality rate; postoperative morbidity rate; 5-year survival rate; and details of postoperative complications such as wound infection, intra-abdominal infection, leakage of hepaticojejunostomy, cholangitis, intra-abdominal bleeding, hepatic failure, pneumonia, and pleural effusion. All data were independently checked by two authors (D. W. and G. W.). Discrepancies between the two reviewers were resolved through discussion or by a third person (Q.W.). The outcomes of surgical resection in 11 studies were extracted and are summarized in Table 1. The details of postoperative complications were characterized in 9 of 11 studies, and the information is presented in Table 2.

**Table 1:** Outcomes of radical resection of HCCA without CLR from 11 studies.

			Resections		R0 (%)		Mortality (%)		Morbidity (%)		5-year survival (%)	
References	Published year	Time period	With CLR	Without CLR	With CLR	Without CLR	With CLR	Without CLR	With CLR	Without CLR	With CLR	Without CLR
Nimura, Y^24^	1990	1979–1989	45	N/A	100 (45)	N/A	6.4 (3)	N/A	41.3 (19)	N/A	40.5 (18)	N/A
Tabata, M^25^	2000	1994–1998	13	12	84.6 (11)	16.7 (2)	7.69 (1)	0.00	30.8 (4)	16.7 (2)	58-3year (7)	0.00
Kawarada, Y^26^	2002	1976–2000	25	N/A	88 (22)	N/A	4 (1)	N/A	20 (5)	N/A	49.9 (12)	N/A
Nagino, M^27^	2005	1999–2003	100	N/A	N/A	N/A	3 (3)	N/A	67 (67)	N/A	N/A	N/A
Nagino, M^28^	2006	N/A	8	N/A	87.5 (7)	N/A	0.00	N/A	25 (2)	N/A	37.5 (3)	N/A
Papoulas, M^29^	2011	2004–2009	15	N/A	80 (12)	N/A	13 (2)	N/A	N/A	N/A	38 (5)	N/A
Lee, J. H^30^	2012	1999–2009	132	N/A	73.48 (97)	N/A	N/A	N/A	N/A	N/A	N/A	N/A
Wahab, M. A^31^	2012	1995–2010	80	79	71.2 (57)	38 (30)	8.8 (7)	3.85 (3)	53.8 (43)	50.6 (40)	28 (22)	5 (4)
Bhutiani, N^32^	2018	2000–2015	90	166	76 (68)	60 (99)	5.6 (5)	6 (10)	59 (53)	66 (109)	N/A	N/A
Juntermanns, B^33^	2019	N/A	24	N/A	N/A	N/A	N/A	N/A	N/A	N/A	48.5 (16)	N/A
Sulai Liu^34^	2020	2015-2018	6	N/A	66.67 (4)	N/A	0.00	N/A	16.67 (1)	N/A	N/A	N/A

**Table 2:** The postoperative complications in HCCA without CLR from 11 studies.

		Resections		Wound infection		Intra-abdominal infection		Leakage of hepaticojejunostomy		Intraabdominal bleeding		Hepatic failure		Pneumonia or pleural effusion	
Author	Published Year	With CLR	Without CLR	With CLR	Without CLR	With CLR	Without CLR	With CLR	Without CLR	With CLR	Without CLR	With CLR	Without CLR	With CLR	Without CLR
Nimura, Y^24^	1990	45	N/A	6	N/A	1	N/A	5	N/A	1	N/A	4	N/A	1	N/A
Tabata, M^25^	2000	13	12	1	1	N/A	N/A	2	0	0	0	1	0	N/A	N/A
Kawarada, Y^26^	2002	25	N/A	1	N/A	N/A	N/A	3	N/A	0	N/A	2	N/A	N/A	N/A
Nagino, M^27^	2005	100	N/A	30	N/A	19	N/A	7	N/A	10	N/A	6	N/A	49	N/A
Nagino, M^28^	2006	8	N/A	N/A	N/A	1	N/A	N/A	N/A	N/A	N/A	N/A	N/A	N/A	N/A
Papoulas, M^29^	2011	15	N/A	4	N/A	N/A	N/A	2	N/A	N/A	N/A	3	N/A	3	N/A
Wahab, M. A^31^	2012	80	79	15	11	14	16	5	5	N/A	N/A	12	9	N/A	N/A
Bhutiani, N^32^	2018	90	166	N/A	N/A	7	17	11	22	N/A	N/A	6	6	N/A	N/A
Sulai Liu^34^	2020	6	N/A	N/A	N/A	N/A	N/A	1	N/A	N/A	N/A	N/A	N/A	N/A	N/A

### Evolution of total CLR in HCCA

The first case of right hemiliver combined with CLR for gallbladder cancer was reported by Cavalcantiti^35^ in 1959. In 1975, Starzl *et al*.^24^ described five cases of right liver lobe combined with right CLR and five cases of left lobe combined with total CLR for hilar tumors at Denver Veterans Hospital; this is the first report of CLR in surgery for HCCA. The principle of CLR depends on the location and degree of invasion. However, he did not delve into the relationship between CLR and the prognosis. In 1986, Mizumoto *et al*.^36^ performed radical resection combined with CLR in 11 patients with HCCA. In 1990, Nimura *et al*.^37^ of Nagoya University School of Medicine reported 46 radical resections of HCCA combined with CLR, and 44 caudate lobes were invaded, which was the first large case of total CLR in HCCA. Routine preoperative percutaneous transhepatic cholangiography or endoscopic retrograde cholangiography (ERC) showed possible caudate bile duct invasion in all cases in this cohort, and Nimura first proposed that HCCA patients with preoperative examination findings or suspected caudate lobe invasion should be combined with CLR. In 1993, Gazzaniga *et al*.^38^ reported 19 patients with HCCA who underwent radical surgery, and recurrence occurred in the caudate lobe in four cases (21%). Ogura *et al*.^39^ reported caudate lobe invasion in 20 cases (36.4%) after 55 radical resections of HCCA. Among these, there were 11 cases of bile duct invasion and 9 cases of direct liver parenchyma invasion. Despite a gradual increase in related reports, the impact of CLR on the prognosis of HCCA has not been conclusive. Until 2000, Tsao *et al*.^40^ retrospectively analyzed the data of HCCA patients undergoing radical resection with or without CLR at the Lahey Clinic in America and Nagoya University in Japan. Among the 25 patients in Lahey, 2 (8%) underwent surgery combined with CLR, and 7 had margin-negative resections, but all patients died within 10 years. However, 109 (89%) of 122 patients underwent radical resection with CLR at Nagoya University, and 96 (79%) underwent R0 resection, with a 10-year survival rate of 14.16%. The rates of surgical death and complications in the two patient cohorts were similar (*P*=0.306 and *P*=0.295, respectively). The results suggested that radical resection combined with CLR could significantly improve the rate of negative resection and the overall survival rate. This study initially clarified the significance of CLR in HCCA treatment.

### Effect of radical surgery in combination with CLR on HCCA

The surgical approach to treat HCCA changed dramatically from 1988 to 2003 at the Academic Medical Center of Amsterdam, the Netherlands. Dinant *et al*.^25^ divided 99 radical resection cases into 3 periods in 5 years. In period 1 (1988–1993), local resections were performed in 41 (91%) patients; in period 2 (1993–1998), 13 (81%) patients who were diagnosed with type III tumors underwent local resections combined with partial liver resection; in period 3 (1998–2003), CLR combined with partial hepatectomy was used to treat HCCA patients with type III and IV tumors (15 cases, 71%). The results showed that the negative margin rate increased from 13% in stage 1 to 59% in stage 3 (*P*<0.05), and the overall survival rate (OS) increased from 33±7% in stage 1 to 60±11% in stage 3 (*P*<0.05), with constant morbidity and mortality. In 2020, Birgin *et al*.^26^ published a meta-analysis reviewing the outcomes of patients after radical resection of HCCA with or without CLR. A total of 1350 cases were included in the study, and the data showed that in the combined CLR and no CLR groups, the median postoperative survival time was 30–64 m vs. 17–35 m, and the disease-free survival was 21–53 m vs. 15–51 m. The R0 resection rate in the surgery with CLR was also better than the surgery without CLR. More importantly, there were no differences in morbidity (RR=0.93; 95% CI: 0.77–1.13; *P*=0.48; *I*^2^=0%) and mortality (RR=1.01; 95% CI: 0.42–2.41; *P*=0.99; *I*^2^=0%) between the two groups.

**Figure 2:**
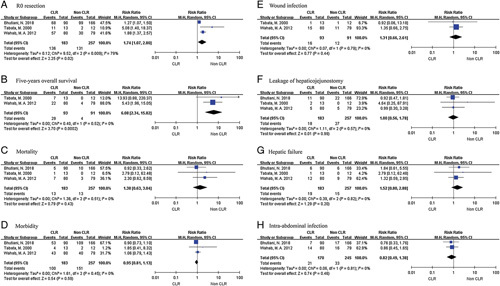
Forest plot of outcomes and complications. A: R0 resection; B: Five-years overall survival; C: Mortality; D: Morbidity; E: Wound infection; F: Leakage of hepaticojejunostomy; G: Hepatic failure; H: Intra-abdominal infection.

Outcomes specific to CLR in the resection of HCCA from 11 studies published during the period 1990–2023 were extracted and summarized in Table 1 ^27–34,37,41,42^. For surgery combined with CLR, the R0 resection rate ranges from 66.67 to 100%, and the overall survival of 5 years ranges from 37.5 to 49.9%. Among them, three articles conducted a detailed study on the outcome of surgery with or without CLR. Studies reporting on the R0 resection rate revealed a significant improvement in patients in the CLR group (RR=1.7; 95% CI: 1.07–2.80; *P*=0.02; *I*^2^=79%). However, these three studies had high statistical heterogeneity, which may be due to differences in the proficiency of surgical procedures in medical institutions. Through separate analyses, it was found that the R0 resection rate in the CLR group was significantly higher than that in the non-CLR group (Fig. 2A). In addition, the 5-year survival rate of the CLR group was significantly higher than that of the non-CLR group (RR=6.08; 95% CI: 2.34–15.82, *P<*0.01; *I*^2^=0%), indicating that the CLR group had a better treatment effect (Fig. 2B). There was no significant difference in mortality and complication rates between the two groups (*P=*0.43, *P=*0.59, respectively) (Figs 2C and 2D).

We further compared the occurrence of six complications between the two groups. Except for some missing data in the non-CLR group, there was no significant difference in the occurrence of complications, such as wound infection, leakage of hepaticojejunostomy, hepatic failure, and intra-abdominal infection (Fig. 2E-H). However, because of the lack of RCTs with large sample sizes, the benefit of radical surgery in combination with CLR requires further evidence.

### Anatomical basis of total CLR

#### The biliary tract and portal vein of the caudate lobe

**Figure 3:**
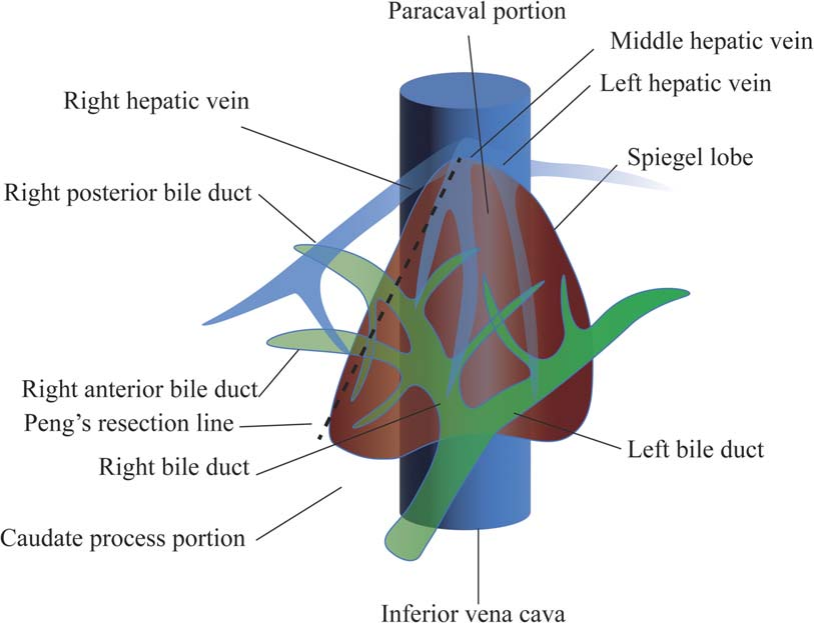
The anatomy of caudate lobe. The caudate lobe is divided into Spiegel lobe, paracaval portion and caudate process portion.

The caudate lobe is divided into the Spiegel lobe, paracaval portion, and caudate process portion^43^. The Spiegel lobe is located on the left side of the caudate lobe, on the left side of the inferior vena cava and the Arantius tube (the umbilical vein ligament), and the dorsal side of the omentum. The paracaval portion is the middle part of the caudate lobe, located on the right side of the Arantius tube, half-wrapped around the inferior vena cava at the abdominal side, up to the root of the secondary porta of the liver. The upper right boundary of the paracaval portion was connected to the right posterior lobe of the liver without an obvious boundary, and the lower right boundary was connected to the caudate process by the right posterior branch of the portal. The caudate process is located in the right part of the caudate lobe, the right side of which can be fused with the right posterior lobe of the liver or is also a free mastoid structure (Fig. 3).

Kumon^44^ used 23 corrosion liver casts to study the bile duct of the caudate lobe. The biliary branches distributed in the Spiegel lobe showed that only into the left biliary tract was observed in 11 livers (47.9%), into the right and left biliary tract in eight livers (34.8%), into the right biliary tract only in three livers (13%), and the other one (4.3%) with unknown direction. Overall, the biliary branches distributed in the Spiegel lobe were dominated by the left branch with an average of 1.86 ducts per liver.

In the paracaval portion, the biliary branches into the left biliary tract in 10 (52.6%) livers, which included nine livers into the left bile duct and one liver into segment 2. In addition, biliary branches joined the right biliary tract in nine livers (47.4%), including five with the right posterior bile duct, three with the right bile duct, and one with the right anterior bile duct.

Although the biliary branches of the Spiegel lobe and the paracaval portion were mainly on the left side, the proportion of bilateral drainage was very high. However, all of the biliary branches in the caudate process portion joined the right biliary tract, including 16 cases (84.2%) in the right posterior bile duct and three cases (15.8%) in the right bile duct (Table 3).

**Table 3:** The biliary branch of caudate lobe.

	Biliary branch	Cases	Rate (%)
Spiegel lobe	Left biliary tract	11	47.9
	Left and right biliary tract	8	34.8
	Right biliary tract	3	13
	Unclear	1	4.3
	Total	23	100
Paracaval portion	Left biliary tract	10	52.6
	Right biliary tract	9	47.4
	Total	19	100
Caudate process portion	Right posterior bile duct	16	84.2
	Right bile duct	3	15.8
	Total	19	100

Considering the anatomical factors, if CLR is required, to achieve a higher R0 resection rate, total CLR is recommended rather than partial CLR, thus improving patient prognosis.

#### The depth of invasion and the length of R0 resection in HCCA

R0 resection is an independent risk factor for HCCA^10,45^. The length of the tumor-free resection margin depends on the infiltration pattern and depth of the bile duct in HCCA. Sakamoto *et al*.^46^ divided HCCA into three infiltration types: mucosal, submucosal intramural, and submucosal extramural layers. Among the 62 HCCA surgical specimens, 28% (17 cases) showed mucosal infiltration. The remaining specimens were submucosal layer: 39% (24 cases) were submucosal-intramural, 17% (11 cases) were submucosal-extramural, and 16% (10 cases) were both layers. The infiltration depth ranged from 0.6 mm to 18.8 mm (mean, 6 mm), and 83% of the cases were less than 10 mm. The incidence of anastomotic recurrence was 18% in cases with tumor-free resection margins less than 2.5 mm, and 10% with margins between 2.5 mm and 5 mm. No anastomotic recurrence occurred when tumor-free resection margins were greater than 5 mm. It was suggested that the infiltration depth of HCCA is usually less than 10 mm, and R0 resection can be obtained if the tumor-free margins are more than 5 mm. Seyama^47^ classified 37 cases of HCCA with R0 resection as group A (13 cases) with a tumor-free margin of more than 5 mm and group B (24 cases) with a tumor-free margin of less than 5 mm. The results showed that the 5-year survival rate in group A was significantly better than that in group B (81.8 vs. 27.4%, *P*< 0.05), but there was no difference between group B and R1 resection, further clarifying the importance of a tumor-free resection margin over 5 mm.

Ebata *et al*.^48^ reviewed the pathological results of 253 HCCA surgical specimens and found that the length of the intramural extension was limited to 10 mm, while superficial extension showed a highly variable range, with an average distance of 10 mm and a maximum of 52 mm. However, among 145 HCCA specimens with or without superficial extension, 129 (89%) had proximal extension and 136 (93.8%) had distal extension confined to within 20 mm. Therefore, for aggressive tumors (mainly intramural extension), the margin is recommended to be 10 mm beyond the macroscopic tumor border; for noninvasive tumors (mainly superficial extension), the margin is recommended to be 20 mm beyond the tumor.

In 2018, Ma *et al*.^49^ reviewed 228 HCCA surgery cases in West China Hospital from 2000 to 2017, and the length of the proximal ductal margin (LPM) was measured. The results showed that the median survival of LPM greater than 10 mm was significantly better than LPM less than or equal to 10 mm (27.99 m vs. 15.01 m, *P<*0.001). Based on the above data, all cases were divided into four groups: LPM less than or equal to 5 mm, 5 mm less than LPM less than or equal to 10 mm, 10 mm less than LPM less than or equal to 20 mm, and LPM greater than 20 mm. The OS of LPM of less than or equal to 5 mm (11.02 m, *P*≤0.05) was significantly worse than that of other groups, and 5 mm less than LPM less than or equal to 10 mm was worse than LPM greater than 20 mm. In addition, preoperative CA-199 levels are closely related to HCCA prognosis^50^. The OS in the LPM greater than 20 mm was significantly better than that in the 10 mm less than LPM less than 20 mm (*P<*0.05) when preoperative CA-199 greater than or equal to 150.0 U/ml. However, because the level of CA-199 was significantly affected by jaundice, more accurate R0 resection criteria need to be further studied in multicenter studies. Overall, an LPM of greater than or equal to 10 mm should be achieved to obtain a survival benefit.

#### The role of lymphadenectomy in HCCA

Lymph nodes are an important prognostic factor of HCCA. In 2010, Ito *et al*.^51^ reported the effect of the number of lymphadenectomies on the postoperative survival of patients with HCCA. They analyzed 144 patients with HCCA who underwent curative-intent resection between 1987 and 2007. They found that patients who underwent R0 resection, based on a total lymph node count less than 11, had a disease-specific survival that was significantly worse than that of patients classified as N0 based on a total lymph node count greater than or equal to 11. In 2015, Kambakamba *et al*.^52^ performed a systematic analysis, suggesting that a lymph node count greater than or equal to 7 is adequate for prognostic staging, while a lymph node count greater than or equal to 15 does not improve the detection of patients with positive lymph nodes. Although many studies have found that the total number of lymphadenectomies performed during radical resection of HCCA seems to be related to the overall survival of patients, a consensus has not yet been reached. Nari *et al*.^53^ evaluated the impact of the number of positive nodes and their relationship to the total number of lymph nodes in 58 HCCA patients. The results suggested that two or more positive nodes were poor prognostic factors. Considering that the smaller the number of lymph node dissections, the less likely it is to have positive lymph nodes, which can affect prognosis, some scholars have proposed using the ratio of positive lymph nodes to the total number of lymph nodes to predict prognosis. Giuliante *et al*.^54^ conducted a retrospective multicenter study of 175 patients who underwent resection for HCCA in 2016. They found positive lymph node ratio exceeding 0.20 was the only independent prognostic factor for overall survival in N1 patients after radical resection. However, this method can only partially reduce the impact of total lymph node dissection on the results. The relationship between the number of lymphadenectomies and prognosis needs to be verified using a large sample from multiple centers.

### Controversy over total CLR of Bismuth type I, II

For Bismuth types III and IV, resection combined with CLR has reached a consensus, but the necessity of Bismuth types I and II is still controversial. Ikeyama *et al*.^55^ reviewed 54 patients with Bismuth types I and II at Nagoya University Hospital from January 1979 to December 2005, whose tumor types were classified as nodular, infiltrating, or papillary by preoperative cholangiography. Among the 31 patients with nodular or infiltrating types, 18 underwent right hepatectomy combined with CLR and the remaining 13 underwent extrahepatic biliary resection or less extended hepatectomy. The R0 resection rates were 100 and 53.8% (*P*=0.0023) and the 5-year survival rates were 62.9 and 23.1% (*P*=0.003), respectively. Seven out of 13 patients diagnosed with Bismuth type I underwent extrahepatic bile duct resection, and only four patients achieved R0 resection. For the papillary type, all eight patients who underwent bile duct resection or less extended hepatectomy resection achieved R0 resection, with a 5-year survival rate of 62.5%. The above results indicate that in patients with Bismuth type I and type II, if tumors are nodular or invasive type, right hepatectomy combined with CLR is recommended if the tumor is papillary type, extrahepatic bile duct combined with less extended hepatectomy resection (according to the resection margin) is adequate^55^, and CLR is not required.

Lim *et al*.^56^ classified 52 Bismuth type I and II patients in 2000–2012 into a combined CLR hepatic lobectomy group (group A, 26 cases) and a bile duct resection alone group (group B, 26 cases). Group A (22 cases, 75%) was mainly Bismuth type II, whereas group B (18 cases, 69%) was mainly Bismuth type I. There was no difference in tumor types between the two groups (21 cases of nodular or invasive and five cases of papillary in group A; 19 cases of nodular or invasive and seven cases of papillary in group B). The results showed that the R0 resection rate in group A was significantly higher than that in group B (100 vs. 73.08%, *P*=0.01), as was the OS rate (*P*=0.047). Moreover, after the removal of the seven cases of R1 resection in group B, the postoperative local recurrence rate in group A was significantly lower than that in group B (11.54 vs. 52.63%, *P*=0.006). There was no difference in postoperative complication rates between the two groups. The above results showed that hepatic lobectomy combined with CLR is more suitable for Bismuth types I and II than for bile duct resection alone^56^. However, this study was not classified according to tumor type, and there was no difference in tumor type distribution between the two groups; the main tumor types were nodular or invasive in the two groups (80 in group A and 73% in group B). Therefore, the results did not conflict with those of a previous study by Ikeyama *et al*.^55^. In summary, the operation for Bismuth type I or type II HCCA should be based on the evaluation of the preoperative tumor type; that is, nodular or invasive type is recommended to perform right/left liver lobectomy combined with CLR, and papillary type is recommended to perform bile duct resection combined with partial liver resection (based on the resection margin).

### Surgical approach of CLR

Due to the extremely complex anatomical location of the caudate lobe, CLR is challenging. The surgical approach for caudate lobe tumors included the left approach, right approach, combined left and right approach, and median approach^57^. All three resection methods separate the short hepatic vein and then remove the hepatic parenchyma; this is called anterograde CLR. Peng Shuyou^58,59^ also proposed retrograde CLR, which is used for caudate lobe tumors close to the inferior vena cava, which are difficult to free from the short hepatic vein, or caudate lobe tumors that are too large to push. Unlike caudate lobe tumors undergoing resection, the CLR of HCCA is performed simultaneously with hepatic lobe resection, which is less difficult than caudate lobe tumor resection alone. Both approaches cut the short hepatic vein of the lateral caudate lobe of the liver first, then cut the liver parenchyma through the median approach, and finally resect the caudate lobe on the unilateral side, while caudate lobe tumors require bilateral resection.

### The resection boundary of the caudate lobe and the right hepatic lobe

The left side of the caudate lobe is free and has an Arantius tube as an anatomical marker^60^, the resection line on the left side is easily determined. However, there is no obvious boundary between the right side (especially the paracaval portion) and the right posterior lobe of the liver, so the right boundary is difficult to judge^61^. Therefore, it was difficult to completely remove the caudate lobe while retaining the right lobe of the liver. Takayama *et al*.^62^ proposed in 1991 that the boundary could be determined by injecting indocyanine green into the right posterior branch of the portal vein to stain the right posterior lobe of the liver, but this is difficult to achieve under laparoscopy. Another method is to establish an ischemic line by ligating all the blood vessels of the caudate lobe during anterograde resection of the caudate lobe of the liver to find the boundary with the right posterior lobe of the liver^63^. In addition, the right posterior branch of the portal vein can be dissected in the Rouviere groove, and the resection margin can be determined by establishing an ischemic line after temporary blocking of the right posterior liver lobe. This method is more practical when the hilar structure is clear. However, HCCA is mostly accompanied by severe jaundice, and some patients do not have a good effect on preoperative jaundice reduction, which also blurs the ischemic line from the liver color. Peng^21^ proposed Peng’s resection line (caudate lobe cut line): one point is the tip of the caudate lobe, the specific location is the angle between the vein ligament, left hepatic vein, and inferior vena cava; the other point is the intersection point between the caudate process and right liver, and the connection between the two points is Peng’s resection line. Overall, when performing right liver lobectomy combined with CLR, CLR is relatively complete, but when performing left liver lobectomy combined with CLR, it is prone to a partial residual caudate lobe.

### Advantages of CLR in laparoscopic

With the development of laparoscopic radical resection of HCCA (LRRHCCA), the combination of laparoscopic liver lobe and CLRs has also been valued^64^. Preliminary clinical practice has shown that, in contrast to open abdominal surgery, LRRHCCA has the following advantages:1. Laparoscopy has a ʻtunnel effectʼ. The removal of the caudate lobe from the foot to the head is more in line with the resection of the visual field. 2. Laparoscopy has a ʻmagnifying effectʼ^65^, which makes it safer to deal with the arteries, portal veins, hepatic veins, variant veins, and bile ducts (such as caudate lobe veins flowing into the right hepatic vein, caudate lobe bile ducts flowing into the right posterior branch, etc.) of the I, II, and III hepatic portals. 3. Laparoscope has a ʻdrilling effectʼ, which makes the space required for suture, ligation, and other operations smaller; 4. Laparoscopy has a ʻradial axis effectʼ, which can perform the ʻleft approachʼ, ʻright approachʼ and ʻmedian approachʼ at the same time, without free and turning the reserved side liver, to reduce the crushing damage to the liver. A multicenter, propensity score-matched study was performed to assess the safety and efficacy of laparoscopic CLR and found that laparoscopic surgery was associated with less blood loss (100 vs. 300 ml, respectively; *P<*0.001) and a shorter postoperative stay (6.0 vs. 8.0 days, respectively; *P=*0.003) compared with open CLR. In addition, all patients achieved R0 resection and the overall morbidity rate was identical in each group^66^. We believe that laparoscopic surgery is a feasible surgical choice for CLR in HCCA patients. In the future, a large multicenter study will be help clarify this issue.

## Discussion

In this study, we systematically reviewed the literature on surgery combined with CLR for the treatment of HCCA and noted that it could significantly improve the rate of negative resection margins, thereby extending the patient’s overall survival time. Meanwhile, combined with CLR in radical resection surgery does not increase perioperative mortality and the incidence of complications, which has been widely adopted for the treatment of Bismuth type III and IV HCCA.

To explore the reasons why total CLR is recommended, many scholars have focused on the anatomy of the caudate lobe. Research^44^ has shown that the bile ducts in the caudate lobe mostly converge into the left and right hepatic ducts rather than one side, and the proportion of bilateral confluence is relatively high. This indicates that the bile ducts of the entire caudate lobe are highly interconnected, and total CLR is recommended rather than partial CLR to achieve a higher R0 resection rate.

The length of R0 resection in HCCA is also important; it depends on the infiltration pattern and depth of the bile duct. A large number of cases^48,49^ showed that for aggressive tumors (mainly intramural extension), the tumor-free resection margins should be at least 10 mm; for noninvasive tumors (mainly superficial extension), the margin should be great than 20 mm, which could help for obtain a survival benefit.

In terms of lymph node resection, there is no consensus on the impact of the total number of lymph node resections, number of positive lymph nodes, and proportion of positive lymph nodes on the prognosis of HCCA patients. Current research^54^ suggests that a positive lymph node ratio exceeding 0.2 may be an independent prognostic factor for overall survival in N1 patients with HCCA. The relationship between the number of lymphadenectomies and prognosis needs to be verified using a large sample from multiple centers.

For Bismuth types I and II, the necessity for resection combined with CLR remains controversial. Current research^55^ suggested that the operation for Bismuth type I or type II HCCA should be based on the evaluation of the preoperative tumor type; that is, nodular or invasive type is recommended to perform right/left liver lobectomy combined with CLR, and papillary type is recommended to perform bile duct resection combined with partial liver resection (based on the resection margin). This surgical concept may be more scientific and effective, but it requires preoperative cholangiography to diagnose clinical pathological types, which is slightly cumbersome in operation, and further research is needed on more convenient and noninvasive diagnostic methods.

Unlike isolated caudate lobectomy, in HCCA radical surgery, caudate lobectomy often requires a combination of left or right hepatic lobectomy. In most cases, when performing right lobe combined with total caudate lobectomy, it is necessary to carefully dissociate the spiegel lobe and separate the caudate lobe along the left approach; when performing left lobe combined with total caudate lobectomy, it is necessary to free the paracaval portion and caudate process portion and separate the caudate lobe along the right approach^57^. Owing to the unclear boundary between the right part of the caudate lobe and the right lobe of the liver, the right boundary of the caudate lobe is difficult to judge. Compared with injecting indocyanine green into the right posterior branch of the portal vein, ligating all the blood vessels of the caudate lobe, Peng’s resection line^21^ (caudate lobe cut line) may be a better choice because it is accurate, easy to identify, and clinically easy to operate. However, when performing left liver lobectomy combined with CLR, it is prone to a partial residual caudate lobe.

Compared to open surgery, laparoscopic radical resection of HCCA has gradually been performed globally because of its enlarged and clear field of view, unique perspective, and minimal trauma. Although laparoscopic surgery requires a large number of surgeons, we believe that this technology can be widely popularized in the future, bringing greater clinical benefits to patients.

## Conclusions

Although radical resection combined with CLR is adopted by most surgeons^67^, it is still a great controversy for HCCA patients with Bismuth type I or II. Right or left hepatic lobectomy combined with CLR is recommended in patients with nodular or invasive tumors. For the papillary type, bile duct resection combined with partial liver resection (based on the resection margin) is sufficient. Bismuth types III or IV should be routinely combined with total CLR, regardless of the presence of invasion manifestations. In addition, a tumor-free margin greater than 10 mm contributes to better survival benefits. LRRHCCA combined with total CLR is more convenient, but the operation time is relatively long and has high technical requirements, which need to be completed by experienced laparoscopic doctors^68^.

## Ethical approval

None.

## Consent

None.

## Sources of funding

This work was supported by grants from the National Natural Science Foundation of China (grant numbers 81974438 and 82173069), Zhejiang Provincial Natural Science Foundation of China (LQ23C060003) and General research program of Zhejiang Provincial Department of health (2023KY124).

## Author contribution

D.W. and Y.C.: study concept and design; D.W., G.W., B.W., and Q.W.: data collection; D.W., F.X., J.C., W.L., and Y.C.: data analysis and interpretation. All authors contributed in writing the paper.

## Conflicts of interest disclosure

All of the authors have no conflicts of interest to declare.

## Research registration unique identifying number (UIN)

We have successfully registered with the name of ʻThe value of total caudate lobe resection for hilar cholangiocarcinomaʼ, and we obtained the UIN on the Research Registry, the number is reviewregistry1684, the hyperlink to the registration is (https://www.researchregistry.com/browse-theregistry#registryofsystematicreviewsmeta-analyses/)

## Guarantor

Yongjun Chen.

## Data availability statement

All data is presented in the text. In addition, all of the data can obtain from the corresponding author.

## Provenance and peer review

Not commissioned, externally peer-reviewed.
